# Modelling the distribution of *Aspalathus linearis* (Rooibos tea): implications of climate change for livelihoods dependent on both cultivation and harvesting from the wild

**DOI:** 10.1002/ece3.985

**Published:** 2014-03-11

**Authors:** Daleen Lötter, David Maitre

**Affiliations:** CSIR, Natural Resources and the EnvironmentP.O Box 320, Stellenbosch, 7602, South Africa

**Keywords:** Bush tea, climate change scenarios, endemic medicinal plants, local communities, MaxEnt, range shifts, rooibos

## Abstract

*Aspalathus linearis* (Burm. f.) R. Dahlgren (rooibos) is endemic to the Fynbos Biome of South Africa, which is an internationally recognized biodiversity hot spot. Rooibos is both an invaluable wild resource and commercially cultivated crop in suitable areas. Climate change predictions for the region indicate a significant warming scenario coupled with a decline in winter rainfall. First estimates of possible consequences for biodiversity point to species extinctions of 23% in the long term in the Fynbos Biome. Bioclimatic modelling using the maximum entropy method was used to develop an estimate of the realized niche of wild rooibos and the current geographic distribution of areas suitable for commercially production. The distribution modelling provided a good match to the known distribution and production area of *A. linearis*. An ensemble of global climate models that assume the A2 emissions scenario of high energy requirements was applied to develop possible scenarios of range/suitability shift under future climate conditions. When these were extrapolated to a future climate (2041–2070) both wild and cultivated tea exhibited substantial range contraction with some range shifts southeastwards and upslope. Most of the areas where range expansion was indicated are located in existing conservation areas or include conservation worthy vegetation. These findings will be critical in directing conservation efforts as well as developing strategies for farmers to cope with and adapt to climate change.

## Introduction

There is compelling evidence of climate change induced impacts on species diversity through among others, species composition changes (Bertrand et al. 2011; Ruiz-Labourdette et al. 2013), range shifts (Bertin 2008; Colwell et al. 2008), and altered phenology (Cleland et al. 2007; Prieto et al. 2009; Hulme 2011). Given the rate and magnitude of changes in the global and regional climate, knowledge of what determines species ranges is critical in understanding the potential consequences for agriculture, forestry, and biodiversity conservation (Araùjo and Rahbek 2006; Falk and Mellert 2011; Bradley et al. 2012). Increasing attention has, therefore, been focussed on implementing a proactive approach through developing plausible scenarios of future climate change and modelling the associated species range and ecosystem shifts.

Decision tools such as correlative spatial distribution models (SDMs) have become key in assessing biodiversity responses to climate change (Midgley et al. 2003; Guisan and Thuiller 2005; Heikkinen et al. 2006; Araùjo et al. 2011; Rodríguez-Castañeda et al. 2012). Several SDM methods have been developed and applied to investigate species' geographic ranges and possible shifts under global climate change. These include mechanistic models, climatic envelope methods, and machine learning techniques (Yates et al. 2010). All of these methods estimate a species actual or potential geographic range through relating field observations of species occurrences to environmental and climatic variables. This relationship can then be used to assess species' range shifts under different climate scenarios to undertake risk assessments in specific focal areas.

In light of the importance of accurately modelling species' responses to a changing climate, numerous studies have been devoted to exploring the relevance, application, and shortcomings of these models (Guisan et al. 2006; Heikkinen et al. 2006; Elith and Leathwick 2009; Soberòn and Nakamura 2009; Miller 2010; Araújo and Peterson 2012). Some cross-cutting objections against these models are as follows: (1) They do not include biotic interactions and assume species distribution is primarily affected only by climatic variables; (2) when extrapolating to the future they make the assumption that the limiting factors and biotic interactions will remain the same; (3) the spatial and temporal resolution at which data are collected and applied raises several statistical issues; (4) while species distribution models usually deal with the mean climatic range of a species potential current and future suitability, it is more often the changes in climatic variability and occurrence of extreme events that determine their distribution range. A prerequisite for distribution modelling is, therefore, a thorough understanding and interpretation of the many factors interacting within the environment where the species occur. Modelling range shifts also requires an in-depth understanding and rigorous analysis of the species at hand. By acknowledging and being aware of the limitations of these methods, we can make them useful support tools exploring climate change associated range shifts.

Globally, numerous species distribution models investigating the impact of climate change on species predict that more species will experience substantial range shifts with a changing climate (Parmesan and Yohe 2003; Thuiller et al. 2005; Broennimann et al. 2006; Chen et al. 2011). Locally, climate change-related species distribution research (Midgley et al. 2002, 2003) in the Cape Floristic Region (CFR) of South Africa suggests a reduction in the geographic ranges of endemic species and reductions in species richness under climate change. The extent of the Cape Fynbos Biome could decline by between 51% and 65% depending on the warming scenario. There is consensus between climate models that the climate in the CFR is expected to become warmer and drier, with a decline in winter rainfall, especially in the western region. This could eventually result in species extinctions of 23% in the Fynbos Biome.

In this study, bioclimatic modelling was employed to model *A. linearis*' distribution. The objectives of the study were to (1) identify the environmental factors limiting or determining the natural distribution; (2) use this to develop a first estimate of the realized niche and potential geographic distribution of wild rooibos and the current geographic distribution of areas suitable for commercially production; (3) inform the location and design of field experiments to assess its ability to survive under different climatic conditions; and (4) develop possible scenarios of range/suitability shift under future climate conditions.

*Aspalathus linearis* is a leguminous shrub indigenous to the Fynbos Biome of the Cape Floristic Region (Dahlgren 1968), which has successfully made the transition from wild resource to an agriculturally important plant. Wild populations of *A. linearis* have a narrow geographic range within the Fynbos Biome and are largely confined to mountain ranges of the far southwestern part of the Northern Cape Province and Cederberg mountains of the Western Cape. The species grows mainly in nutrient poor, highly acidic and well-drained, sandstone-derived soils (pH 3–5.3) typical of the mountainous areas in the area (Muofhe and Dakora, 2000). Its climatic distribution is dictated particularly by the combination of winter rainfall and hot dry summers with an annual rainfall of at least 300–350 mm (Dahlgren 1968). Cultivated and wild *A. linearis* differ mainly in terms of morphology, growth, and flowering patterns (Malgas et al. 2010). Cultivated plants are reseeders, whereas certain ecotypes of wild *A. linearis* are slower growing resprouters. Rooibos is ant dispersed and its fire-stimulated seeds germinate in the early winter months after the passage of the first rain bearing cold fronts. Ant dispersal provides a number of benefits for the species. Ants may move seeds many meters away from the parent plant helping it to escape from herbivores and minimizing competition with parent plants/siblings (Bond and Slingsby, 1983). In commercially propagated rooibos, these critical stages of seed germination and seedling emergence are artificially overcome by sowing seed in well-prepared, irrigated seedbeds after which it is then removed and established in plantations.

**Figure 1:**
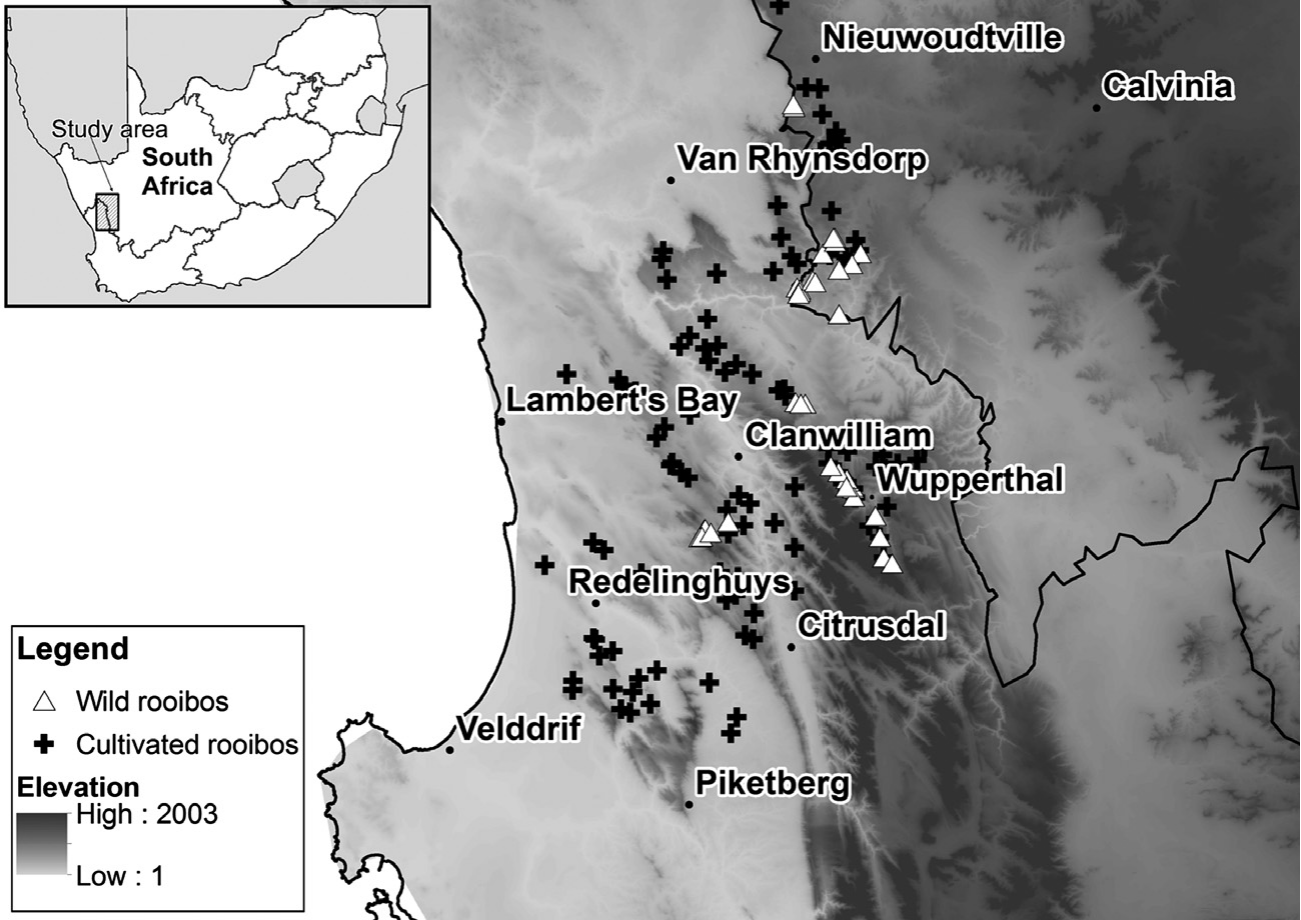
Map of the study area: surveyed locations of wild and cultivated rooibos stretching from Nieuwoudtville in the Northern Cape, south toward Piketberg.

The species was first described circa 1768, but wild plants have been collected and utilized by local inhabitants of the Cederberg and Bokkeveld mountains (Fig. 1) for centuries (Morton 1983). Based on rock art and archaeological evidence, hunter-gatherers have lived in the area for 10–20,000 years and herders (Khoi) since around 1200 AD (Barnard 1992). Rooibos has always formed an integral part of the heritage of these people, and they have a rich knowledge of managing and utilizing the plant to produce tea as well as for its medicinal and health properties. The economic value of rooibos was, however, not exploited until the 1930s when intensive research on the cultivation of the plant enabled the development of the full-fledged industry as it stands today. The industry is one of the largest providers of permanent and seasonal employment in the rural areas of South Africa (Department of Agriculture, Forestry and Fisheries 2010). Recent years have seen an unprecedented growth in the rooibos industry as the demand for rooibos from international markets has steadily increased.

Alongside the well-established commercial rooibos industry, traditional small-scale farming fulfills a vital role in maintaining the economic and social stability of historically neglected rural communities in the semiarid Cederberg region of South Africa. Small-scale farmers are concentrated in isolated and remote rural areas around Nieuwoudtville and Wupperthal. Wild rooibos is marketed by these small-scale farmers as an organic and fair trade certified product to niche markets overseas. Many rural communities therefore depend on *A. linearis* for their livelihoods, so the tea has ecological, economic, and cultural significance.

## Data and Methods

### MaxEnt

The maximum entropy method is a robust model widely applied in the field of ecology (Elith and Leathwick 2009) and similar to Poisson point process models (Renner and Warton 2013). The MaxEnt software (version 3.3.3k http://www.cs.princeton.edu/~schapire/maxent/) was used in this study to model species geographic distributions. It is a machine learning technique that uses a data matching algorithm to make predictions from incomplete information (Phillips et al. 2006). Subject to known constraints, MaxEnt estimates a probability distribution that has the maximum entropy while matching the value of each environmental variable as closely as possible to the empirical values observed at the species' occurrence records. The output can be either raw (relative occurrence rate), cumulative, or logistic (probability of presence). There is a debate about whether MaxEnt output should rather be presented in the raw format as opposed to the more widely used logistic format, which relies on postprocessing assumptions (Royle et al. 2012; Merrow et al. 2013; Hastie and Fithian, 2013; Yackulic et al. 2013). As this issue is still unresolved, and suitability is easier to interpret, this study presented the results as logistic output which is an estimate of the suitability (scaled from 0 to 1) of each grid cell within the study area as a function of the values of the climatic and environmental variables in that grid cell. Based on known occurrences of rooibos in the area that it actually occupies, MaxEnt therefore gives an approximation of the suitability for the species, which approximates its realized environmental niche. It therefore fits the model in an environmental space, which is a conceptual area defined by climatic and environmental variables, and then projects it back to geographic space (Pearson 2007).

### Species occurrence data

Presence only locations for both cultivated and wild rooibos were obtained separately. Seventy-one presence location records for wild types of *A. linearis* were assembled during field work and from the literature (Van der Bank et al. 1995; Van Heerden et al. 2003). Distribution data supplied by the South African National Biodiversity Institute's PRECIS database (Germishuizen and Meyer 2003) indicate a natural distribution of rooibos stretching from Nieuwoudtville south toward Cape Town and even Bredasdorp in the southern Cape. The southeasternmost occurrences were not included in the final dataset as they were only available at a quarter degree resolution and could therefore not be used to provide accurate information on the values of the environmental variables at the collection locality. This is further complicated by the significant variation, which exists in climate and soils over short distances due to the heterogeneous and undulating character of the environment. Some locations as far east as Bredasdorp are also thought to be misidentifications (B.-E. van Wyk, pers. comm., 2011). Only those records of wild rooibos (Fig. 1) that could be reliably confirmed and located were used. These locations correspond with the mountain areas of Nieuwoudtville and Wupperthal where abundant wild populations occur and most of the wild species harvesting is carried out by the small-scale farmers and, thus, where the study is focussed.

Intensive cultivation of rooibos occurs on the mountain slopes and on top of plateaux or plains in the mountains of the greater Cederberg region. The southernmost production area is the Piketberg, and it extends northwards as far as Nieuwoudtville. One-hundred and one presence records for cultivated rooibos that represent point localities (midpoints of field boundaries) were used as input to develop the current distribution map for commercial rooibos. These were treated separately in the modelling process as they almost certainly represent an artificially modified distribution.

### Environmental variables

A list of variables appropriate for modelling rooibos was obtained from previous studies of species distributions within the Fynbos Biome (Midgley et al. 2002; Malgas et al. 2010) as well as from farmers' knowledge of the variables limiting rooibos distribution. In her study of abiotic and biotic parameters as drivers of *A. linearis'* environmental suitability, Gérard (2010) concluded that rooibos is mainly driven by abiotic factors and indicates climate as a limiting aspect of its distribution. There is a strong correlation between elevation, temperature, and rainfall in the study area, and hence, the following climatic variables were used as input for the MaxEnt model: total winter rainfall, total summer rainfall, average winter minimum temperatures, and average summer maximum temperatures. In a winter rainfall climate, rooibos regeneration will require sufficient soil moisture during the winter months for seed germination and establishment, while some precipitation during the summer season is necessary to enable young seedlings to survive through the dry summer months. This guided the choice of variables relating to seasonal rainfall. The wild plants' altitudinal distribution lies between 450 m and 900 m above sea level. Allowing for potential elevation shifts brought on by climate change, minimum and maximum temperatures were included. The climate variables were also chosen to correspond to variables, which could be obtained for future climate scenarios. Baseline climate data representing interpolations of observed data for the time period 1950–2000 were obtained from WORLDCLIM (Hijmans et al. 2005) in ESRI grid format at a resolution of 30 arc-sec. Topography, soil depth, and drainage are other important factors affecting the establishment of rooibos plantations and distribution of wild populations. Slope and land type were therefore also included as predictor variables. Slope was derived from a 90 m digital terrain model (Jarvis et al. 2008a,b2008b). Land types were obtained from the Agricultural Research Council's Institute for Soil, Climate, and Water (SIRI 1987). Land types describe the unique combination of macroclimate, terrain form (i.e., location on a catena), and soil pattern as determined by the underlying geology and weathering patterns.

A winter rainfall region was delineated as background for the modelling process as rooibos is known to occur in an area where winter rainfall during May–September accounts for 60% or more of the total rainfall for the year. There are no known records of rooibos occurring in a bimodal or summer rainfall region. All of the surveyed locations are thus captured by this background. This criterion centers the focus on the region where the activities of local farmers could potentially be impacted on by climate change. The selected background extends well beyond the areas where rooibos presently occurs and includes adequate environmental space to quantify low suitability as well as allowing for possible range shifts. All the environmental variables were converted to a 30 sec grid by resampling using the nearest neighbor method.

### Model building

The selected species occurrence records and environmental variables were used to develop a model of the potential distribution of wild and cultivated rooibos under current climatic conditions. The MaxEnt algorithm was first run with presence locations of wild rooibos. The rooibos presence records were randomly assigned so that 75% of the localities were used for training data, and the remaining 25% were reserved for testing the model. The MaxEnt algorithm was run with hinge and quadratic features with several combinations of the environmental variables. The model was run with different sets of the training data where after a different fraction of the data were withheld for each run. Variables were then narrowed down to the best combination based on the contribution that each variable, as well as all variables collectively, made to the bioclimatic envelope. Response curves also gave an indication of the dependence of the predicted suitability on a specific variable as well as the range under which the variable reaches its optimum suitability. The output was projected as a map of species distribution showing the suitability on a scale from 0 (least) to 1 (most) suitable. To discriminate between truly “suitable” and “unsuitable” areas, a threshold of occurrence was chosen to correspond to the lowest predicted suitability for a species occurrence record following Pearson (2007).

To assess wild rooibos species range adjustment in the face of climate change, the model for the current conditions was projected using an ensemble of scenarios under potential climate change. A distribution map for each scenario was developed and further analyzed in ArcGIS to establish areas of overlap between the different projections. A model average was then calculated from the suite of projections and compared with the existing species distribution map to quantitatively assess range expansions or shifts. These maps were based on the assumption that species are allowed unrestricted dispersal to new areas that satisfy their climatic limits (Guisan et al. 2006; Loarie et al. 2008).

### Model evaluation

The model needs to be assessed to determine how well the model fits the training data and predicts the current distribution of the species. As noted by Elith and Graham (2009), this is a semisubjective process and different methods of performance can be applied. Among others, variable importance was assessed by observing the change in gain when certain variables were excluded. All maps and functions were also visually evaluated for irregularities. Specific features have been made available in the latest version of MaxEnt to test model appropriateness and fit when projecting to a new environment. The multivariate environmental similarity surface (MESS maps) supplied with the software was used to show whether the portion of the predicted range is within the environmental space defined by the ranges of the variables in the input to the model.

### Climate scenarios

To assess rooibos species range adjustment or shift in the face of climate change, the underlying model structure that has been developed under current conditions was applied under potential scenarios of future climate change. An ensemble of models that assume the A2 emissions scenario was used to explore rooibos suitability under future conditions. This emission scenario was selected because it reflects high energy requirements and the current actual trajectory of emissions are already ahead of the higher end emissions scenarios. Five coupled climate models, which were dynamically downscaled for Southern Africa by means of the conformal-cubic atmospheric model (CCAM; Engelbrecht et al. 2011), were obtained from the climate modelling and environmental health research group at the CSIR. These simulations were used to generate future changes in temperature and precipitation data by adding the mean differential (Hewitson 2003) between future scenarios (2041–2070) and the baseline (1960–1990) for each climate model to the corresponding observed climate variable.

## Results

### Current climate: cultivated tea

**Figure 2:**
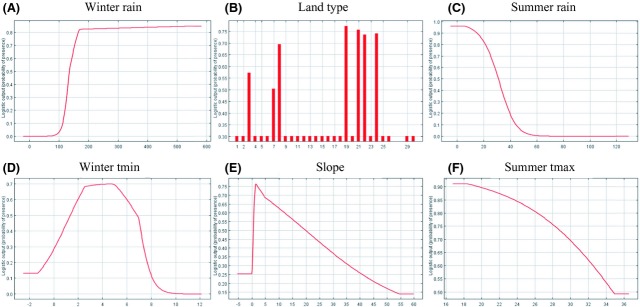
Marginal response curves of the most important predictor variables explaining cultivated tea suitability: (A) average winter rainfall, (B) land types, (C) average summer rainfall, (D) average minimum winter temperature, (E) slope derived from a digital terrain model, and (F) average maximum summer temperature.

The MaxEnt output indicates that the model was statistically significant (*P* < 0.0001) and performed relatively well in predicting suitable crop areas with a test omission rate of 0.08 at the minimum training presence. The variables that made the largest contribution to explaining crop suitability, based on the “jacknife” procedure in MaxEnt, are winter precipitation (45.3%), soil types (28.2%), and summer precipitation (14%). The strong influence of land types indicates that the sandy, infertile soils derived from Table Mountain sandstone origin are required for rooibos production. The marginal response curves for cultivated tea are indicated in Figure 2. The response curve for winter rainfall was open-ended, indicating increasing suitability with increasing winter precipitation (Fig. 2A). The opposite is true for summer precipitation that showed decreasing suitability as rainfall increased (Fig. 2B). However, when summer rainfall was excluded from the model, winter precipitation displayed a bell-shaped response curve, indicating reduced suitability as rainfall increases above a certain threshold value. Minimum temperature during the coldest months made a significantly greater contribution to the model than mean annual temperature. The optimum temperature (between 2°C and 6°C) corresponds to an elevation range in mountainous areas at which there is a comparatively high winter rainfall.

**Figure 3:**
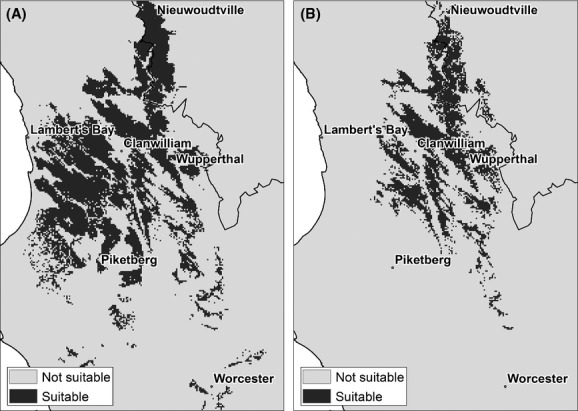
Suitability maps for (A) cultivated and (B) wild *Aspalathus linearis* for the current climate.

The districts of Piketberg, Clanwilliam, Van Rhynsdorp, and Calvinia are known to be key cultivation areas where the unique microclimate and soil combine to form a rather limited geographic area, which is suitable for rooibos production. The final suitability map (Fig. 3A) provides a good representation of these existing core production areas. It also indicates that some small areas in the mountains to the south of the traditional production area are marginally suitable.

### Current climate: wild tea

**Figure 4:**
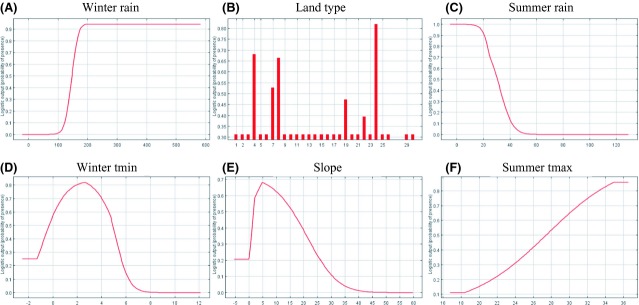
Marginal response curves of the most important predictor variables explaining wild tea suitability: (A) average winter rainfall, (B) land types rainfall, (C) average summer rainfall, (D) average minimum winter temperature, (E) slope derived from a digital terrain model, and (F) average maximum summer temperature.

The potential distribution of *A. linearis* in geographic space (Fig. 3B) was predicted with reasonable success (test omission rate of 0.07) at the minimum training presence threshold. It exhibits a good match to the known distribution of *A. linearis* in the greater Cederberg region (Fig. 3B). The environmental variables used to define the bioclimatic envelope of *A. linearis* corresponded with the variables used in modelling cultivated tea, but differed in the contribution each variable made to define the envelope. According to the jacknife procedure, winter precipitation (31.2%), minimum temperatures (26%) during winter, and land types (18.6%) were the most important variables. Summer precipitation remained a significant variable, although to a lesser extent than for the cultivated tea. Land types of sandstone origin were still significant and also included soils with more rocky outcrops. In addition, slope increased a little in importance with an optimum between 3° and 10°. Both winter and summer precipitation displayed the same open-ended response curves as cultivated tea (Fig. 4A and B). They displayed a similar response of decreased suitability as rainfall increases above a certain value, indicated by the response curves generated using only the corresponding variable. The potential distribution of wild tea is similar to that of the cultivated tea, although more restricted to mountainous areas. The environmental space was also somewhat different. This can be seen in the different optima of the environmental variables and the shape of the response curves (Fig. 4). Minimum temperature during winter exhibits a narrower range of temperatures, which corresponds to the narrower elevation range of wild compared with the cultivated tea.

### Extrapolation to future climate

**Figure 5:**
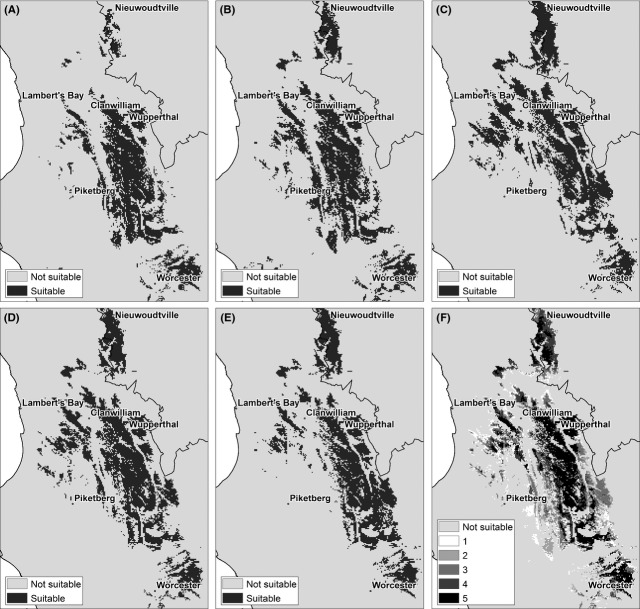
Suitability of areas for cultivated *Aspalathus linearis* under five future climate change scenarios for the period 2041–2070: (A) UKMO, (B) MPI, (C) MIROC, (D), GFDL (E) CSIRO, (F) all models. Map (F) shows the measure of agreement among models on a scale of 1–5 where 5 indicates the strongest overlap.

**Figure 6:**
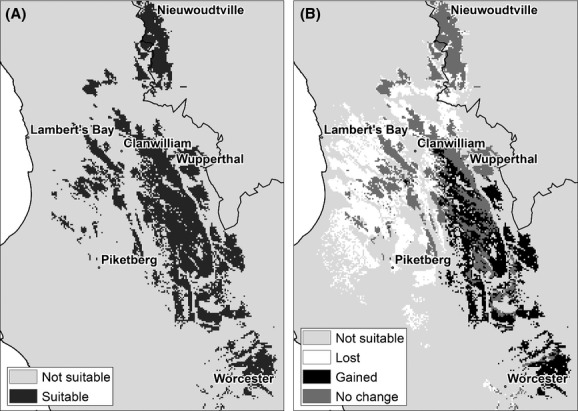
Suitability of areas for cultivated *Aspalathus linearis* under future climate change for the period 2041–2070: (A) average of five climate scenarios, (B) associated range shifts. Map (B) shows areas where range contraction, no change and range expansion occur relative to the current climate (1960–1990).

A suite of five models were applied to model suitability under altered climate conditions (Fig. 5A–E). All the climate models consistently predicted an average increase of 2.7–3.2°C in annual temperatures across the region of interest. Projections for precipitation among models are more variable. The UKMO and MPI model projected the most significant decreases in winter precipitation, whereas the MIROC model mostly predicted increases in winter precipitation. Winter precipitation anomalies therefore ranged from decreases of 52 mm to increases of 32 mm. This resulted in a suite of envelopes of suitability changes depending on the climate model in question. The MIROC model consistently yielded the most conservative predictions because it projects less drastic temperature increases and some winter precipitation increases. There was some overlap in the predicted areas between the models (Fig. 5F). The model ensemble average (Fig. 6A) for an intermediate future period (2041–2070) indicates rooibos tea suitability will remain the same in the higher elevation areas of the traditional rooibos production area. In the western areas along the coast, considerable decreases are however projected, especially in the lower lying regions. Across all scenarios the most significant increases in suitability are expected in the mountainous region toward the south of the study area, indicating a general shift southwards and to higher altitudes (Fig. 6B).

**Figure 7:**
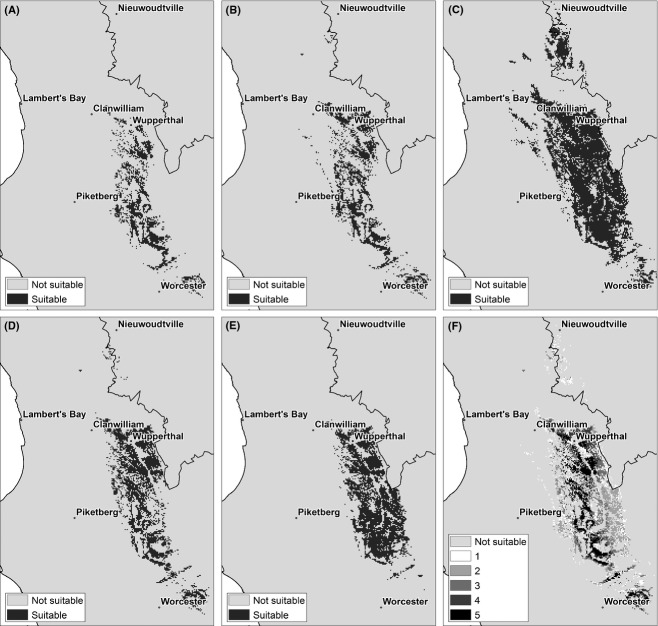
Suitability of areas for wild *Aspalathus linearis* under five future climate change scenarios for the period 2041–2070: (A) UKMO, (B) MPI, (C) MIROC, (D), GFDL (E) CSIRO, (F) all models. Map (F) shows the measure of agreement among models on a scale of 1–5 where 5 indicates the strongest overlap.

**Figure 8:**
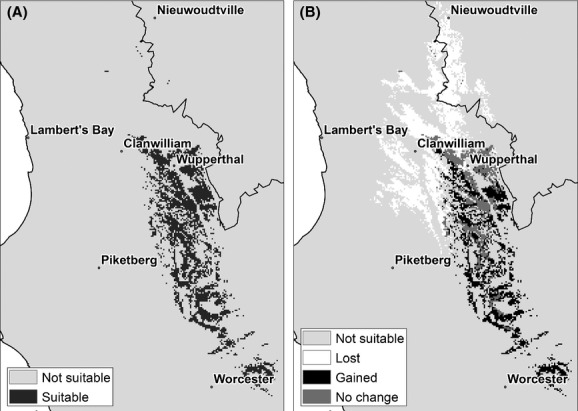
Suitability of areas for wild *Aspalathus linearis* under future climate change for the period 2041–2070: (A) average of five climate scenarios, (B) associated range shifts. Map (B) shows areas where range contraction, no change, and range expansion occur relative to the current climate (1960–1990).

The same suite of climate models was used to model the potential distribution of wild *A. linearis* (Fig. 7A–E) for an intermediate future scenario (2041–2070). Each projection is quite different, and the extent of agreement is less than for the cultivated rooibos (Fig. 7F). The distribution map (Fig. 8A) shows a marked contraction in its bioclimatic range in the northern part of the study area as opposed to the cultivated tea. The Suid-Bokkeveld small-scale farmer community is located in this region and is one of the most important areas where wild rooibos is currently harvested. A further significant range contraction is also visible along the western parts of the study area at lower elevations. These are the areas most vulnerable to species loss. Further south and to the eastern part of the study area, another key harvest locality is found in the region of Wupperthal. Most of this region is not expected to undergo any range shifts (Fig. 8B). Areas that were not predicted to undergo range shifts under future conditions are restricted to elevation ranges of between 800 m and 1050 m above sea level. Range expansion, however, is noticeable toward the south especially along the mountain ranges. This depends on the ability of the species to colonize new sites. Overall, a similar trend of range shift southwards and to higher elevations is observed for both wild and cultivated tea.

## Discussion

### Model scenarios of rooibos crop and wild type distribution

Predictor variables defining rooibos' distribution in the study area were mostly similar for both wild and cultivated tea but differed in their relative contributions to the envelope. The climatic variables mainly pertained to summer precipitation, winter precipitation, and minimum temperatures during winter. The findings agree with those of Hawkins et al. (2011) which indicated elevation and rainfall as best predictors of rooibos species distributions. The MaxEnt response curves suggest that even limited increases in summer rainfall will negatively affect the predicted suitability of areas for rooibos. Farmers have however reported that rainfall during summer, which is mainly brought about by thunderstorms, is particularly important for rooibos persistence. The response functions further indicated that rooibos suitability increases as winter rainfall increases above 150 mm. Lastly, the responses to minimum temperature during winter indicated that minimum temperatures exceeding 5.8°C negatively affect predicted suitability for rooibos. The MaxEnt model predicted *A. linearis* to occur mainly in mountainous areas with well-drained sandstone and quartzitic soil types and is therefore consistent with ecological knowledge of the species. This potential distribution under current conditions is comparable (yet somewhat more restricted in some areas), to the distribution of wild rooibos developed by Malgas et al. (2010), which may be due to the absence of soil information in their analysis. Although the model indicated both cultivated rooibos as well as its wild relatives tend to prefer similar habitats, the cultivated rooibos had a significant greater range.

The relative difference in distribution between the wild and cultivated types can mainly be attributed to the aid of mechanization, soil manipulation practices, and transplanting seedlings into fields. Hence, cultivated rooibos can be grown on a much larger scale and over a wider geographic range than the natural distribution, thus expanding the limits of its range. This often results in the cultivation of rooibos in marginal areas where the climatic conditions are outside the limits of its natural range. The use of seedlings rather than sowing seed to establish new plantations helps the plant through the critical stages of germination and seedling establishment, which is known to be an important determinant the distribution of populations in the landscape (Harper 1967; Harper and White 1974; Clark et al. 2007;. Commercial cultivation is thus not limited by germination and establishment requirements to the same extent as wild tea and therefore more widely distributed.

Over the western parts of southern Africa, the temperatures are projected to increase at about twice the global rate, while winters are projected to become drier (Engelbrecht, 2005). Increased temperatures and decreased rainfall, especially during critical stages of rooibos growth and development, may have serious implications for the sustainability of rooibos production in certain areas. The projected shifts in the distribution and suitability found in this study under future climates are comparable to those found in other studies of endemic plants (Midgley et al. 2003; Lenoir et al. 2008) and in crop suitability (Bradley et al. 2012). The direction of range shifts for both wild and cultivated tea is generally southeastwards and upslope. This means that lowlands on the west coast will first experience climate change impacts, whereas higher altitude mountain areas will experience little if any impacts for the period 2041–2070. Similarly, Loarie et al. (2008) projected that more species are likely to persist in mountain areas or expand their ranges to higher elevations. These novel areas might be viable for cultivation as propagation and plantation establishment is supported by human intervention. However, most of the areas where range expansion is indicated are located in existing conservation areas or include conservation worthy vegetation. The ability of wild rooibos to successfully migrate to these novel areas is uncertain. It will depend on complex interactions between abiotic and biotic variables, the species ability to disperse, population size, and regeneration strategies (Midgley et al., 2003). This will require further investigations that fall outside the scope of this study.

### Implications for livelihoods, conservation, and adaption planning

Emerging, small and resource poor farmers are often particularly vulnerable in that they do not have sufficient resources and access to timely information to deal with adverse effects of climate change. The bulk of wild tea is harvested by small-scale farmers located in near-pristine natural environments around Nieuwoudtville and Wupperthal. Small-scale farmers harvest proportionally less wild tea in relation to the cultivated variety, yet income per tonne from these teas is substantially higher and a valuable commodity. If species' ranges shrink or shift in the future as is predicted by the models, it is doubtful whether farmers will relocate to areas where species have colonized new sites. More pressure might be placed on harvesting the remaining populations and may contribute to the species decline. Research regarding the direction and rate of species distribution shifts is therefore important for local nongovernmental organizations engaging with small-scale farmers in developing strategies to cope with and adapt to climate change.

Land use change driven by the massive expansion of the rooibos industry in recent years has led to extensive habitat loss of many indigenous and endemic species including *A. linearis* (Raimondo et al. 2009). The total rooibos crop footprint in this global biodiversity hot spot is currently 79,000 ha (Pretorius 2009). Some of these locations in the lowland areas of the production area are already marginal for commercial rooibos cultivation. Existing climate variability exerts pressure on sustainable production, and these areas may be the first to experience the effects of climate change. The ability to cultivate tea in areas where it did not occur naturally may also threaten genetic integrity of different wild types and cause homogenization of the species (Van der Bank et al. 1995). Future projections of crop suitability shifting toward mountain catchment areas are therefore a cause of concern and knowledge of future distribution patterns in the landscape will be critical in directing conservation efforts. Such information will aid the Rooibos Biodiversity Initiative which has become an important instrument in regulating land clearance and promoting sustainable land management practices.

Globally, numerous medicinally and economically useful endemic plants contribute significantly to the well-being and cultural heritage of indigenous communities. Many of these medicinal plant species are however under pressure due to unsustainable resource exploitation and degradation of habitats, while the additional challenges posed by climate change could drive some species to extinction (Cavaliere 2009; Gairola et al. 2010; Gaikwad et al. 2011; Ray et al. 2011). Similarly, habitat destruction and climate change pose a significant threat to rooibos' future survival. Rooibos tea is a treasured South African commodity, creating many employment opportunities and inextricably intertwined with the heritage and traditions of the local communities. Timely management intervention and adaptation to climate change is therefore of paramount importance to protect wild rooibos populations and sustain commercial production.
